# Programming the lymph node microenvironment to enhance anti-tumor T cell immunity in neuroblastoma

**DOI:** 10.1186/2051-1426-3-S2-P434

**Published:** 2015-11-04

**Authors:** Joshua M Gammon, Christopher M Jewell

**Affiliations:** 1 Fischell Department of Bioengineering, University of Maryland, College Park, MD, USA

## Background and hypothesis

Current cancer therapies rely on nonspecific chemotherapies which cause severe side effects without combating relapse. Therapeutic cancer vaccines aim to harness the adaptive immune response to specifically target and eliminate established tumors, while generating durable tumor-specific T cell responses to prevent relapse. Toll like receptor agonists (TLRas) such as CpG strongly activate dendritic cells (DCs) and show promise as adjuvants for cancer vaccines when co-administered with tumor associated antigens (TAAs). Current strategies are exploring direct vaccination with TLRas and TAAs to prime DCs *in vivo*, but are hampered by the instability of vaccine components, inefficient co-localization of antigen and adjuvant, and poor trafficking and persistence in lymph nodes (LN) - tissues that orchestrate adaptive immunity. Direct LN injection of soluble vaccines show enhanced efficacy over systemic administration, but are limited by rapid flushing of vaccine components. Biomaterials offer the potential to enhance cancer vaccination by allowing sustained release, co-delivery, and protection of encapsulated cargo. We recently demonstrated that local delivery of non-toxic, degradable biomaterials loaded with antigens or adjuvants potently enhances antigen specific T cell immunity. Building on this work, we hypothesized that local introduction of particles loaded with CpG and soluble tumor lysates (TLs) might combat tumor progression in murine neuroblastoma models.

## Methods and results

Degradable vaccine depots consisting of Poly(Lactide-co-Glycolide) microparticles encapsulating CpG (3.45±0.37 µg of CpG per mg of polymer) were synthesized by double emulsion. For prophylactic studies, mice were primed by intra lymph node (i.LN) injection at the inguinal lymph nodes using vaccine depots suspended in lysates prepared from murine neuroblastoma cells (Neuro-2a). Mice were boosted two weeks later subcutaneously at the tail base with soluble vaccine components. One week after the boost, lymphocytes from peripheral blood were pulsed with TL and stained intracellularly for IFNg by FACS. 0.71±0.13% of CD8^+^ T cells from mice immunized with vaccine depots secreted IFNg compared to 0.38±0.1% from the untreated group. Two weeks after the boost mice were challenged with 10^6^ N2a cells. Strikingly, while 75% of mice in the unvaccinated group reached defined endpoints based on tumor burdens by day 15, 75% of mice vaccinated i.LN with vaccine depots were tumor free at day 20. Ongoing studies are comparing efficacy of i.LN vaccine depots to leading clinical adjuvants, and investigating therapeutic treatment regimens. These data demonstrate a potential new route for harnessing biomaterial vaccine carriers to program the LN environment to help combat pediatric cancer.

